# Urogenital schistosomiasis and associated determinant factors among senior high school students in the Dutsin-Ma and Safana Local Government Areas of Katsina State, Nigeria

**DOI:** 10.1186/s40249-016-0158-1

**Published:** 2016-08-02

**Authors:** Tolulope Ebenezer Atalabi, Umar Lawal, Francis Omowonuola Akinluyi

**Affiliations:** 1 Department of Biological Sciences, Faculty of Science, Federal University, Dutsin-Ma, P.M.B. 5001, Dutsin-Ma, Katsina State Nigeria; 2 Department of Remote Sensing and GIS, School of Earth and Mineral Sciences, Federal University of Technology, Akure, Ondo State Nigeria

**Keywords:** Prevalence, Intensity, Urogenital schistosomiasis, Risk─factors, Katsina State, Nigeria

## Abstract

**Background:**

Human schistosomiasis is a chronic parasitic disease of poverty caused by the cercariae of digenetic trematodes of the genus *Schistosoma*. The disease is a major source of morbidity and mortality in 77 low- and middle-income countries in the tropics where 700 million people are at risk. In a bid to provide relevant epidemiological information to boost control of urogenital schistosomiasis at the state level in Nigeria, we conducted this study with the aim of investigating the disease’s prevalence and intensity, and the determinant factors responsible for its endemicity.

**Methods:**

Data on risk factors were obtained from a total of 645 students aged 12─25 years using well- designed questionnaires. Samples were collected between 09:45 and 14:00 in universal bottles. Each10μl centrifuged sample was examined for the eggs of *S. haematobium* using Motic® (Binocular) Light Microscope (model S-10-P) with a x10 objective. Average infection intensity was recorded as number of eggs per 10 ml of urine sample. Survey data were entered into Microsoft Excel 2010 and analyzed using Epi Info™ 7. Associations among variables were determined using the chi square test and bivariate and multivariate logistic regressions.

**Results:**

Prevalence of urogenital schistosomiasis was 30.54 % among the study population, with a mean infection intensity of 30.27 eggs/10 ml of urine. Prevalence and average intensity were higher in males (28.37 % and 32.21 eggs/10 ml of urine respectively) than in females (2.17 % and 5 eggs/10 ml of urine respectively). Water contact activities (*X*^*2*^ *= 29.031, P = 0.0000*), sex (male) [*X*^*2*^ *= 109.82; P<0.0001*], location (Dutsin─Ma) [*X*^*2*^ *= 7.19; P = 0.0073*], age group 18-20 (*X*^*2*^ *= 4.819, P = 0.0281*), altitude (531─560 m) [*X*^*2*^ *= 6.84, p = 0.0089*], fathers doing other brown─collar jobs (*X*^*2*^ *= 8.449, P = 0.0037*) and mothers’ occupation (*X*^*2*^ *= 9.470, P = 0.0021*) were found to be significantly associated with urogenital schistosomiasis. Boys were six times more likely to be infected with the cercariae of *S. haematobium* compared to girls [A*OR* (95 % *CI*): 6.34 (4.89─8.22)].

**Conclusions:**

Dutsin-Ma and Safana were classified as moderate-risk Local Government Areas for urogenital schistosomiasis. The strong association between the disease and mother’s occupation is of utmost importance and suggests a promising control measure: that is, directing health education as well as grassroots mass chemotherapeutic intervention with praziquantel at mothers. A good network including treated pipe-borne water, drainage system, and sewage disposal facilities available should be improved upon. Molluscicides should be provided at highly subsidized rate to help control the disease.

**Electronic supplementary material:**

The online version of this article (doi:10.1186/s40249-016-0158-1) contains supplementary material, which is available to authorized users.

## Multilingual abstracts

Please see Additional file 1 for translations of the abstract into the six official working languages of the United Nations.

## Background

Human schistosomiasis is a chronic parasitic disease of poverty caused by the cercariae of digenetic trematodes of the genus *Schistosoma*. Also referred to as snail fever, the disease came into the limelight of public health research in 1851, when it was discovered by the German surgeon; Theodor Bilharz in Egypt [1]. Being a major source of morbidity and mortality in 77 low- and middle-income countries in Africa, Asia, the Caribbean, Middle East, and South America, where 700 million people are at risk, the disease is now the second most devastating and dreaded tropical disease in the world after malaria [2–4]. From a total of 207 million global cases, 120 million are reportedly symptomatic and 20 million are severe [2].

In Africa, about 176 million people from 46 countries are reportedly infected with the disease while an alarming 280 000 have been estimated to die annually [5].

Nigeria has been described as the most endemic country for schistosomiasis in Sub-Saharan Africa, with 29 million people reportedly infected and an estimated 101.3 million people at risk of infection [6].

An increase in the population density in locations where access to potable water is limited creates a need for alternative water sources, which can be unwholesome. Consequently, communities situated near infested lakes, rivers, ponds and streams, risk getting a severe infection. Studies have shown that the distribution of infection among endemic communities is lopsided with 5─10 % often being heavily infected [7]. Age, sex, altitude and mean annual rainfall have been identified as important risk factors for the transmission of the disease [8, 9]. Domestic chores, irrigation, and recreational activities, coupled with adventure-driven behaviors, mean that school-aged children frequently come into contact with infested water bodies, thereby; making them more at risk of contracting the disease [10]. To corroborate this, a report has shown that 45.93 % of the global population requiring chemotherapeutic interventions with praziquantel is school-aged children [4].

In a bid to provide relevant epidemiological information to boost control of urogenital schistosomiasis at the state level in Nigeria, we conducted this study with the aim of investigating the disease’s prevalence and intensity, and the determinant factors responsible for its endemicity.

## Methods

### Study area

The survey was carried out in the Dutsin-Ma and Safana Local Government Areas (LGAs) of Katsina State, North West Nigeria. The study area covers a total surface area of 809 km^2^ and is inhabited by 353 450 people, according to the 2006 National Census [11]. In Dutsin-Ma, the duration of rainfall is May to September with annual mean of 700 mm. It has a temperature range of 29─31 °C [12]. Meanwhile, Safana, a neighboring LGA to Dutsin-Ma, has a similar duration of rainfall.

The ethnic groups comprise the Hausa and Fulani, who are predominantly farmers and traders. The study area has a short grass type of savanna and is located at the extreme North of Nigeria.

### Study design

The cross-sectional survey was carried out between May and August, 2015. It was designed to target a total number of 645 senior high school students from the Dutsin-Ma and Safana LGAs.

### Inclusion and exclusion criteria

Students in the age group of 12─25 years voluntarily enrolled in the study. However, respondents who demonstrated unwillingness were not included in the study.

### Sample size and sampling technique

Six schools were drawn from a list of 20 senior high schools in both LGAs. Sample size was allotted to each school, and by extension, each class of participating students. Six hundred and forty-five students were selected using a simple random sampling technique from a total of 3 537 students. The schools selected for the study were: the Government Pilot Senior Secondary School, Safana; the Government Senior Secondary School, Tsaskiyya; the Community Day Senior Secondary School, Safana; the Government Pilot Senior Secondary School, Dutsin-Ma; the Community Day Senior Secondary School, Dutsin-Ma; and the Government Senior Secondary School, Dutsin-Ma.

### Data collection

#### Socio-demographic and risk factors

Well-designed questionnaires were used to collect data on socio-demographic and determinant factors. A school-based questionnaire was administered to each principal and was used to collect data on students’ population, history of praziquantel distribution, reports of haematuria, and local languages for haematuria (blood in urine) and *Bulinus* species. Individual questionnaires were used to collect information peculiar to each respondent, while a urinalysis form was used to document urine parameters.

#### Urine sample collection

Clean 20 ml plastic health diagnostics universal bottles with screw-caps were used to collect different quantities of terminal urine samples between the hours of 9:45 and 14:00 [13]. This coincides with the periodicity of egg excretion in *S. haematobium-*infected respondents [14].

#### Centrifugation of urine samples

Test-tubes each containing about 5 ml of urine was loaded into a C2 series Centurion Scientific centrifuge (United Kingdom). Spinning was performed at 2 250 revolutions per minute for 1.5 minutes. The supernatant fluid was decanted off, while the egg-containing sediment was dissolved with a small portion of the supernatant fluid to enhance clarity when viewing the wet mount under the microscope.

#### Microscopic examination of urine for *S. haematobium* eggs

Each 10 μl sample was pipetted onto a grease-free glass slide using an adjustable micro-pipette (10 μl─100 μl) and covered at an angle with a glass slip to avoid bubble formation. Examination of samples for *S. haematobium* eggs was carried out using Motic® Binocular Light Microscope (Chna), model S-10-P, with a x10 objective. For positive samples the eggs were counted and each average count was recorded as number of eggs per 10 ml of urine [15]. Intensity of infection was categorized as light (< 50 eggs/10 ml of urine) or heavy (≥50 eggs/10 ml of urine) [16].

#### Quality control

Universal sample bottles had corresponding serial numbers. Urinalysis of each sample was carried out within 90 seconds of inserting dipstick. Depending on whether infection intensity was heavy or light, the number of *S. haematobium* eggs in each sample was counted between two and seven times with the average intensity recorded.

#### Statistical analysis

Survey data were entered into Microsoft Excel 2010 (USA) and analyzed using Epi Info™ 7 (Atlanta, USA). Associations between variables were determined using the chi square test, bivariate and multivariate logistic regressions. Strength of associations was measured using odds ratio (OR) at 95 % confidence intervals (CIs). Crude OR was adjusted using haematuria, a morbidity marker. A *P─value* of less than 0.05 was considered to be statistically significant.

#### Ethical approval

Written ethical permission to conduct the study was given by the Ethical Committee of the Katsina State Post-Primary Education Zonal Office, Dutsin-Ma. School heads and students were briefed about the aims of the study. They orally consented to participate in the study. Information from the respondents was kept confidential. The results of the study were communicated to the school heads, and mass drug administration, in consultation with the state Ministry of Education, was recommended.

## Results

### Socio-demographic characteristics of the respondents

Of the total 645 students enrolled in the study, 405 (62.79 %) were males and 240 (37.21 %) were females. The mean age (standard deviation, SD) of the respondents was 16.79 (1.97) years. The percentage of students in each age group was as follows: 12─14 (7.91 %), 15─17 (59.69 %), 18─20 (28.37 %), 21─23 (3.72 %) and 24─26 (0.31 %). White-collar (42.48 %) and brown-collar (56.12 %) jobs were the major occupation categories of the respondents’ fathers, while the mothers’ occupations were mostly white-collar and brown-collar jobs and housewives, representing 10.23 %, 52.56 % and 36.90 % of the respondents respectively (see Table 1).

**Table 1:** Socio-demographic features of respondents from Safana and Dutsin-Ma LGAs

Variables	Frequency	Percentage (%)
Age		
12–14	51	7.91
15–17	385	59.69
18–20	183	28.36
21–23	24	3.73
24–26	2	0.31
Sex		
Male	405	62.79
Female	240	37.21
Father’s occupation		
White collar job	274	42.48
Farming	113	17.52
Other brown collar jobs	249	38.60
Late	6	0.93
Retiree	3	0.47
Mother’s occupation		
White collar job	66	10.23
Farming	1	0.16
Other brown collar jobs	338	52.40
Late	2	0.31
House wifery	238	36.90
Total	645	100%

### Risk factors associated with urogenital schistosomiasis

Of the 645 students surveyed, 228 (35.35 %) said they had previously swum in water sources, while 303 (46.98 %) previously played in shallow water. Bore-hole, dams, ponds, rivers, streams, wells, taps and sachets were indicated as sources of water for domestic use by 448 (33.81 %), 227 (17.13 %), 43 (3.25 %), 38 (2.87 %), 184 (13.89 %), 148 (11.17 %), 231 (17.43 %), and six (0.45 %) respondents respectively (see Table 2).

**Table 2:** Frequency of risk factors associated with Urogenital schistosomiasis from Safana and Dutsin-Ma LGAs

Variables	Frequency	Percentage (%)
Swimming experience		
Yes	228	35.35
No	417	64.65
	645	
Playing in shallow water		
Yes	303	46.98
No	342	53.02
	645	
Sources of water for drinking,		
Cooking, washing & bathing.		
Bore hole	448	33.81
Dam	227	17.13
Pond	43	3.25
River	38	2.87
Stream	184	13.89
Well	148	11.17
Tap	231	17.43
Sachet	6	0.45
Total	1 325	100.00

NOTE: Respondents combined multiple water sources for domestic use

This informed the higher total number above compared to the 645 interviewed

### Prevalence and intensity of urogenital schistosomiasis by study location

**Fig. 1:**
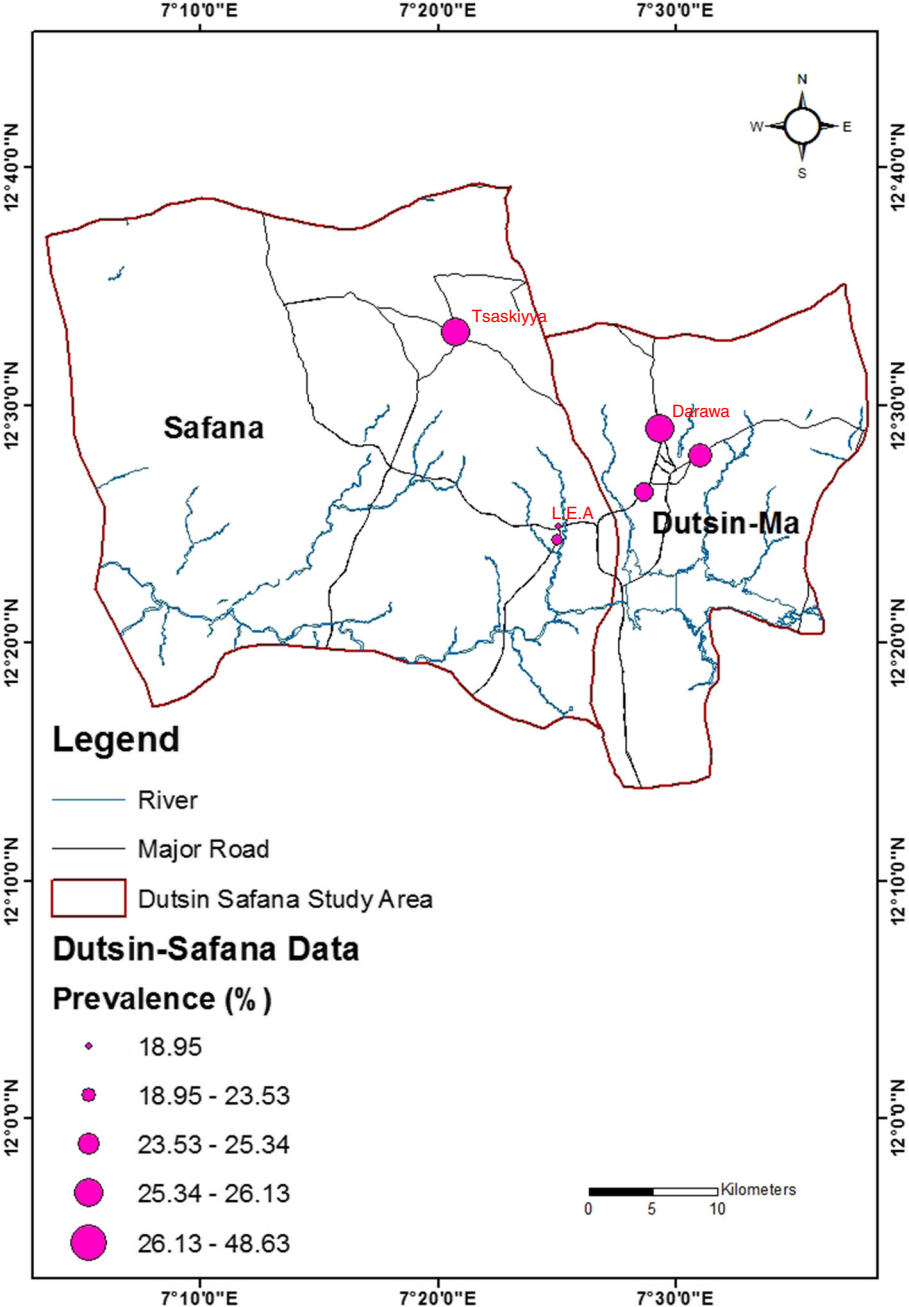
Map of the study area showing the prevalence of urogenital schistosomiasis by study location

The highest prevalence of urogenital schistosomiasis (48.63 %) was recorded in Darawa in the Dutsin-Ma LGA, with the second highest prevalence (46.15 %) recorded in Tsaskiyya, Safana LGA. The lowest prevalence rate (18.95 %) was recorded in a Local Government Education Authority location in Safana (see Fig. 1).

**Fig. 2:**
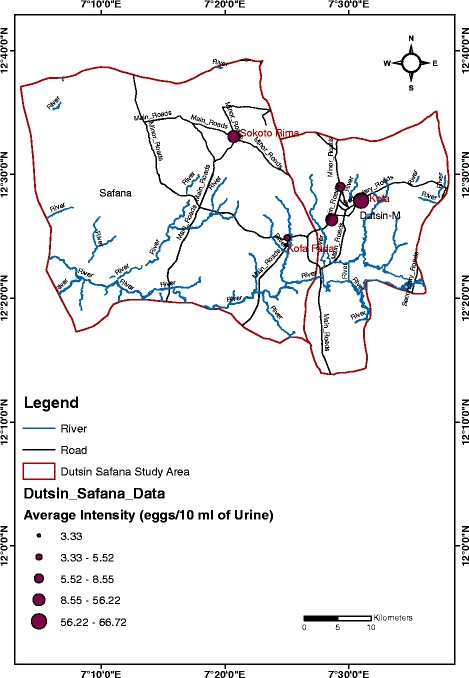
Map of the study area showing the mean intensity of urogenital schistosomiasis infection by study location

Meanwhile, the average infection intensities by location was a bit different in pattern with the highest infection intensity (66.72 eggs/10 ml of urine), and the second highest infection intensity (56.22 eggs/10 ml of urine) recorded in the Kofa and Sokoto Rima communities respectively in Dutsin-Ma LGA. The lowest infection intensity (3.33 eggs/ 10 ml of urine) was recorded in the Kofa Fada community in Safana LGA (see Fig. 2).

### Prevalence and intensity of urogenital schistosomiasis in relation to age and sex

In this survey, the prevalence of urogenital schistosomiasis was 30.54 % with a mean (SD) intensity of 30.27 (50.03) eggs/10 ml of urine. Of the 645 urine samples examined, 197 (30.54 %) tested positive for *S. haematobium* eggs, with a total raw egg count of 5,933 (see Table 3).

**Table 3:** Prevalence and intensity of urogenital schistosomiasis in relation to age and sex

	N.E	N.I	Prevalence (%)	Egg count (Mean)
				95 % *CI*
Sex				
Boys	405	183	28.37	5 863[32.21(19.01-45.42)]
Girls	240	14	2.17	70 [5(-1.21-11.21)]
*P* value				<0.0001
Age				
12–14	51	16	2.48	760[47.5(-35.63-130.63)]
15–17	385	106	16.43	3 546[33.77(15.14-52.41)]
18–20	183	67	10.38	1 562[23.31 (11.93-34.69)]
21–23	24	7	1.09	62[8.86(0.03-17.69)]
24–26	2	1	0.16	3(3)
*P* value				0.0220
Total	645	197	30.54(27.04-34.28)	5 933[30.27(17.97-42.57)]

NOTE: Prevalence rate for each determinant factor was calculated by using the number infected as the numerator while the total number of those interviewed in the survey (645) served as the denominator.

*N.E* Number Examined

*N.I* Number Inspected

*C.I* Confidence Interval

**Fig. 3:**
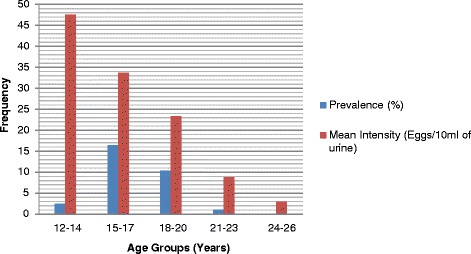
Age group-related prevalence and intensity of urogenital schistosomiasis in the study LGAs

The prevalence and mean egg count (intensity) was higher in males [28.37 % (Chi square test = 109.82, *P<0.0001*) and 32.21 eggs/10 ml of urine)] than in females (2.17 % and 5 eggs/10 ml of urine). The highest prevalence (16.43 %) was recorded among children in the age group of 15─17 years (Chi square test = 0.7505, *P* = 0.687) compared to the 12─14, 18─20, 21─23 and 24─26 age groups with prevalence rates of 2.48 %, 10.38 %, 1.09 %, and 0.16 % respectively. However, as shown in Fig. 3 and Table 3, the highest mean infection intensity (47.5 eggs/ 10 ml of urine) was recorded among the age group of 12─14 years (Chi square test = 22.3149, *P = 0.324*) compared to 15─17, 18─20, 21─23, and 24─26 age groups with mean infection intensities of 33.77, 23.31, 8.86, and 3 eggs/10 ml of urine, respectively.

### Prevalence and intensity of urogenital schistosomiasis in relation to water sources

The highest infection rate (75 %) was recorded among children who used all the open, potentially infested water sources, for domestic purposes, followed by a rate of 55.56 % recorded among subjects who played in shallow water and swam in the water sources. Meanwhile, the lowest prevalence (30.31 %) was recorded among respondents who relied on bore-hole, tap, sachet, or well (closed, potentially un-infested water sources) for domestic use. This value (30.31 %) as well as the mean infection intensity (31.39 eggs/10 ml of urine) for this category, is of major concern.

**Fig. 4:**
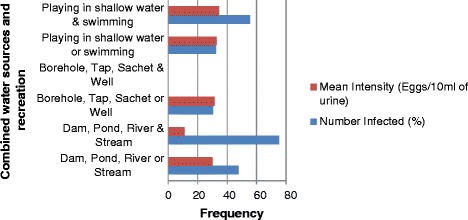
Water source-related prevalence and intensity of urogenital schistosomiasis in the study LGAs

The mean infection intensity was highest (34.29 eggs/ 10 ml of urine) among those who swam and also played in shallow water, followed by those who either played in shallow water or had a history of swimming (32.83 eggs/10 ml of urine). The lowest mean intensity of infection in respondents who depended on all open water sources (dams, ponds, rivers and streams) was found to be 11 eggs/10 ml of urine (see Table 4 and Fig. 4).

**Table 4:** Frequency and intensity of urogenital schistosomiasis in relation to combined water sources for domestic and recreational uses

Water sources	N.E	N.I	Odds Ratio	ECAM	
(Combined)	(%)	(%)	(95 % *CI*)	(95 % *CI*)	*P* value
Dam, Pond, River or Stream	346 (100)	165 (47.69)	2.09 (1.59-2.76)	29.89 (17.45-42.34)	<0.0001
Dam, Pond, River & Stream	8 (100)	6 (75.00)	6.89 (1.38-34.49)	11 (-0.67-22.67)	0.0196
Borehole, Tap, Sachet or Well	617 (100)	187 (30.31)	1 (Reference)	31.39(18.52-44.27)	-
Borehole, Tap, Sachet & Well	0	0	0	0	-
Playing in shallow water or swimming	191 (100)	62 (32.46)	1.11 (0.78-1.57)	32.83 (3.46-62.19)	0.6359
Playing in shallow water & swimming	171 (100)	95 (55.56)	2.87 (2.03-4.07)	34.29 (14.91-53.68)	<0.0001

*ECAM* Egg Count Arithmetic Mean

In the bivariate analysis, the combinations of using dams, ponds, rivers, or streams [C*OR* (95 % CI): 2.09 (1.59─2.76)], dams, ponds, rivers, and streams [C*OR* (95 % CI): 6.89 (1.38─34.49)] and playing in shallow water and swimming [C*OR* (95 % CI): 2.87(2.03─4.07)] were found to be significantly associated with frequency of infection with urogenital schistosomiasis (see Table 4).

Generally, water contact activities were strongly linked with the transmission of the disease (Chi square test =29.0312, *P<0.0001*).

### Prevalence and intensity of urogenital schistosomiasis in relation to the LGA, infection category, and occupation of respondents’ parents

Of the total number of infected respondents, 175 (88.83 %) fell into the light intensity category, while 22 (11.17 %) belonged to the heavy intensity category. Out of the infected respondents, 59(29.95 %) and 138 (70.05 %) lived in the Safana and Dutsin-Ma LGAs, respectively. Similarly, of the 5, 933 *S. haematobium* eggs recovered, 4,792 eggs were recorded in Dutsin-Ma (mean = 34.72 eggs/10 ml of urine), while 1,141 eggs were recorded in Safana (mean = 19.34 eggs/10 ml of urine). Invariably, Dutsin-Ma recorded a higher prevalence and mean intensity of urogenital schistosomiasis (see Table 5).

**Table 5:** Prevalence and intensity of urogenital schistosomiasis in relation to LGAs, category of *S. haematobium* infection and parental occupation of respondents

	N.E	N.I	Prevalence (%)	Egg Count [Mean]
				95 % *CI*
Safana	243	59	9.15	1 141 [19.34(-2.03-40.71)]
Dutsin-Ma	402	138	21.39	4 792 [34.72(19.88-50.08)]
*P* value			0.0073	
Infection category				
Light (≤ 50 eggs/10ml)	175	175	27.13	1 456 [8.32(6.82-9.91)]
Heavy (≥50 eggs/10ml)	22	22	3.41	4 477 [203.5(121.14-285.86)]
Father				
White Collar Jobs	274	71	11.01	1 270 [17.89(0.19-36.09)]
Farming Other brown	113	32	4.96	1 192 [37.25(0.62-73.89)]
Collar jobs	249	94	14.57	3 471 [36.93(18.41-55.44)]
Late (Dead)	6	0	00.00	0
Retiree	3	0	00.00	0
*P* value			0.3516	
Mother				
White Collar Jobs	66	12	1.86	248 [20.67(1.90-39.43]
Farming	1	0	00.00	0
Other Brown Collar Jobs	338	121	18.75	5 007 [41.38(22.13-61.32)]
Late (Dead)	2	1	00.16	6 (6)
House wifery	238	63	9.77	672 [10.67(4.53-16.81)]
*P* value			0.0021	
Total	645	197	30.54	5 933 [30.27(17.97-42.57)]

**Fig. 5:**
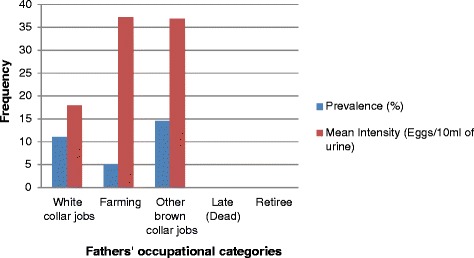
Father’s occupation-related prevalence and intensity of urogenital schistosomiasis in the study LGAs

**Fig. 6:**
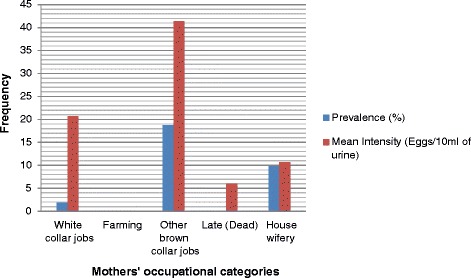
Mother’s occupation-related prevalence and intensity of urogenital schistosomiasis in the study LGAs

In the father’s occupation category, the highest prevalence (14.57 %) of urogenital schistosomiasis was recorded among children whose fathers did “other brown-collar jobs” (mechanics, vulcanizers, cleaners, labourers, etc.) while the highest mean intensity (37.25 eggs/10 ml of urine) of infection was identified among children whose fathers were “farmers” (see Table 5 and Fig. 5). Similarly, in the mother’s occupation category, children whose mothers did “other brown-collar jobs” had the highest prevalence (18.75 %) of urogenital schistosomiasis and associated mean egg count (41.73 eggs/10 ml of urine) [see Table 5 and Fig. 6].

### Bivariate and multivariate analyses of factors associated with urogenital schistosomiasis

In the bivariate analysis, sex (male) [C*OR* (95 % *CI*): 13.32 (7.64─24.39)], location (Dutsin-Ma) [C*OR* (95 % *CI*): 1.63 (1.27─2.09)], and altitude [C*OR* (95 % *CI*): 1.57 (1.12─2.22)] were found to be significantly associated with the prevalence of urogenital schistosomiasis. In the father’s occupation category, only the variable “other brown-collar jobs” [C*OR* (95 % *CI*): 1.73 (1.27─2.36)] was found to be significantly associated with *S. haematobium* infection. The variables “other brown-collar jobs” [C*OR* (95 % *CI*): 2.48 (1.91─3.27)] and “house wives” [C*OR* (95 % *CI*): 1.62 (1.15─2.27)] as the mother’s occupation, were also found to be significantly associated with the disease (see Table 6). In the multivariate logistic regression analysis, only sex (*P<0.0001*) was found to be statistically associated with the disease.

**Table 6:** Multivariate analysis of the variables associated with the frequency of urogenital schistosomiasis among respondents

Variables	Frequency of Urogenital Schistosomiasis			C*OR* (95 % *CI*)	A*OR* (95 % *CI*)
	Subtotal	Positive (%)	Negative (%)		
Sex					
Boys	405 (100)	183 (45.19)	222 (54.81)	13.32 (7.64-24.39)	6.34 (4.89-8.22)
Girls	240 (100)	14(5.83)	226 (94.17)	1	1
Chi Square				109.82	43.82
*P* value				<0.0001	<0.0001
Age					
12–14	51 (100)	16 (31.37)	35 (68.63)	1.20 (0.63-2.25)-	
15–17	385 (100)	106 (27.53)	279 (72.47)	1	1
18–20	183 (100)	67 (36.61)	116 (63.39)	1.52 (1.04-2.21)	1.06 (0.68-1.66)
21–23	24 (100)	7 (29.17)	17 (70.83)	1.08 (0.41-2.64)-	
24–26	2 (100)	1 (50.00)	1 (50.00)	2.62 (0.07-102.99)-	
Chi Square				2.66	
*P* value				0.1032	
Address					
Dutsin-Ma	402 (100)	138 (34.33)	264 (65.67)	1.63 (1.27-2.09)	2.31 (1.76-3.03)
Safana	243 (100)	59 (24.28)	184 (75.72)	1	1
Chi Square				7.19	12.89
*P* value				0.0073	0.0003
Altitude					
509–529m	307 (100)	78 (25.41)	229 (74.59)	1	1
530–558m	338 (100)	119 (34.91)	219 (65.09)	1.59(1.14-2.24)	1.11 (0.75-1.66)
Chi Square				7.257	0.274
*P*-Value				0.0071	0.6008
Father					
White Collar Jobs	274 (100)	71 (25.91)	203 (74.09)	1	1
Farmers	113 (100)	32 (28.32)	81 (71.68)	1.13 (0.69-1.84)	-
Other Brown Collar Jobs	249 (100)	94 (37.75)	155 (62.25)	1.73 (1.19-2.52)	1.97 (1.40-2.77)
Late (Dead)	6 (100)	0 (00.00)	6 (100.00)	0	-
Retiree	3 (100)	0 (00.00)	3 (100.00)	0	-
Chi Square				0.87	
*P* value				0.3516	
Mother					
White Collar Jobs	66 (100)	12 (18.18)	54 (81.82)	1	1
Farmers	1 (100)	0 (00.00)	1 (100.00)	0	-
Other Brown Collar Jobs	338 (100)	121 (35.79)	217 (64.21)	2.51 (1.29-4.87)	1.89 (1.39-2.56)
Late (Dead)	2 (100)	1 (50.00)	1 (50.00)	4.50 (0.26-77.14)	-
House Wives	238 (100)	63 (26.47)	175 (73.53)	1.62 (0.81-3.23)	-
Chi Square				9.485	
*P* value				0.0021	
Grand Total	645 (100)	197 (30.54)	448 (69.46)		

*COR* Crude (Unadjusted) Odds Ratio

*AOR* Adjusted Odds Ratio (indicated only for values with *p*≤0.05)

Boys were six times more likely to be infected with the cercariae of *S. haematobium* compared to girls [A*OR* (95 % *CI*): 6.34 (4.89─8.22)]. Respondents in the Dutsin-Ma LGA were twice [A*OR* (95 % *CI*):2.31 (1.76─3.03)] as likely to suffer infection due to *S. haematobium*. Similarly, students whose fathers’ occupations belonged to the “other brown-collar jobs” category were about two times [A*OR* (95 % *CI*): 1.97 (1.40─2.77)] more likely to be infected than those whose fathers’ occupations belonged to the “white collar jobs” category.

Similarly, respondents whose mothers’ occupation belonged to the “other brown-collar jobs” category were about two times [A*OR* (95 % *CI*): 1.89 (1.39─2.56)] more likely to be infected than those whose mothers’ occupation belonged to the “white collar jobs” category (see Table 6).

## Discussion

Several studies on urogenital schistosomiasis among pre-school children, school-aged children, and adults have been conducted, yet little data exist about urogenital schistosomiasis in senior high school students. Often, findings on this category, where available, have been merged with other groups of school-aged children. This study showed a prevalence of 30.54 % (27.04─34.28 %) of urogenital schistosomiasis among senior high school students in the study area. Expectedly, due to the focal nature of the infection, this result is different from previous findings of 26.8 % in Ebonyi State, South East Nigeria, 52.8 % at the point -of- care in northeastern Zimbabwe, and 44.3 % in North Central Nigeria [17–19]. The differences observable among these prevalence rates might be linked to ecological factors, cultural practices, and water contact activities unique to our study population. This may require further investigation.

The mean intensity of infection was 30.27(17.97-42.57) eggs/10 ml of urine in this study. Previous records from Anambra State, South East Nigeria (10.1 eggs/ 10 ml of urine), Kaduna State, North West Nigeria (73.93 eggs/10 ml of urine) and the Republic of Chad (<13.5 eggs/10 ml of urine) further confirm the focal nature of urogenital schistosomiasis [20–22]. These differences might be as a result of seasonal variations, proximity of infested water bodies, natural tendency of subjects towards recreation (swimming and playing in shallow water), and the state of social amenities in both the study area and some states in Nigeria mentioned above.

A higher prevalence rate of urogenital schistosomiasis (28.37 %) and a higher mean intensity of infection (32.21eggs/ 10 ml of urine) were recorded among males. This conforms to the usual trend recorded previously [23–25] and could be explained by males having more frequent contact with water due to swimming, fishing, molding bricks from clay, and irrigation and construction work.

However, there are contrary reports from Ogun State, Nigeria (Males: 57.1 %, Females: 59.2 %) and Khartoum North, Sudan (Males: 22 %, Females: 34 %) where females suffered a higher prevalence rate of urogenital schistosomiasis [14, 26]. This might be explained by gender-sensitive cultural or religious beliefs unique to ethnic groups which predispose a particular gender to a higher infection rate in the aforementioned areas. For instance, in the northern part of Nigeria, school-aged males fetch water from open sources for sale in a bid to make ends meet.

Generally, the results of this survey reflected that the prevalence rate of urogenital schistosomiasis and associated infection intensity reduced sharply as age increases. This is in agreement with previous findings in Nigeria and Sudan [23, 26] and is possibly due to the cultural practice of according dignity to older children by exempting them from domestic activities that require contact with unwholesome water sources. Access to knowledge about the epidemiology of urogenital schistosomiasis through Health Science and Biology offered as subjects might also contribute to this. In addition, the older people get, the more possible it is to acquire knowledge about disease prevention and control. Conversely, previous studies from Senegal and Ethiopia have reported increases in prevalence of urogenital schistosomiasis as the age of the respondents increased [13, 27], which could possibly be explained by the children in these studies showing an increase in water contact activities as they grow older.

Moreover, the highest prevalence rates in this study were recorded in the age groups of 15─17 (16.28 %) and 18─20 (10.38 %) years. This implies, on a general note, that these adolescents engage in more water contact activities than the other age groups in this survey. It could be further deduced that they shoulder the responsibility of providing water for their homes. The lowest prevalence rate (0.16 %) and mean intensity of infection (3 eggs/10 ml of urine) was recorded in the 24─26 age group. However, the highest mean intensity (47 eggs/10 ml of urine) of infection was recorded in the age group 12─14 years, which is suggestive of more contact with streams, rivers, ponds and lakes while swimming and sourcing water for domestic needs.

In agreement with these findings, it was reported that the highest mean intensity of urogenital schistosomiasis was recorded in the age group of 10─14 years in Plateau State, North Central Nigeria and Ebonyi State, South East Nigeria [23, 28].

Other studies carried out in Plateau and Ogun States, Nigeria reported that the highest prevalence rates were observed in the age group of 15─19 years [25, 29]. Findings from Ebonyi State also reported that the age group above 24 years showed the lowest mean intensity of infection with urogenital schistosomiasis [28].

Also noteworthy is the fact that respondents were reliant on multiple sources of water for domestic consumption. Combined recreational activities of swimming and playing in shallow water [C*OR* (95 % *CI*): 2.87 (2.03─4.07)] using dams, ponds, rivers and streams [C*OR* (95 % *CI*): 6.89 (1.38─34.49)]; and using dams, ponds, rivers or streams [C*OR* (95 % *CI*): 2.09 (1.59─2.76)] were significantly associated with urogenital schistosomiasis.

Respondents who relied on dams, ponds, rivers and streams had the highest frequency of infection (75 %), while those who combined swimming with playing in shallow water bodies recorded the second highest infection rate (55.56 %) and the highest mean intensity of infection (34.29 eggs/10 ml of urine). These are reflections of a high frequency of exposure of the respondents to infested open water bodies. However, a survey conducted in Malawi [30], found that play/bath in an open water body was not found to be associated with urogenital schistosomiasis infection [C*OR* (95 % *CI*): 1.21 (0.35–4.10)]. This suggests that interactions among determinant factors play a significant role in the transmission of the disease.

Furthermore, this survey found that the second highest mean intensity of infection (32.83 eggs/10 ml of urine) was recorded among subjects who either played in shallow water or had a previous history of swimming. A survey carried out in Kaduna State indicated that users of pond water had the highest prevalence rate urogenital schistosomiasis [21], while in Northern Ghana and Blantyre, Malawi, contact with streams, wells, rivers, streams, dams, and springs was found to be associated with *S. haematobium* infection [30, 31]. It is pertinent to recall that the values of disease prevalence (30.31 %) and mean intensity of infection (31.39 eggs/10 ml of urine) obtained among users of bore-holes, taps, sachets and wells appear outrageously high, and there are different perspectives on this. One reason could be due to respondents largely utilizing open, infested water sources, although well water, has the highest tendency of being exposed to the environment compared to other closed water sources. On the other hand, it could be hypothesized that the wells in the study area were shallow and either completely uncovered or partially covered, thus enhancing interaction with *S. haematobium egg*-infested dust particles.

In addition, modern waste disposal facilities are limited and the terrain of the study area is dusty and rocky. Consequently, the soil is stony and it is usually very tasking to dig deep while constructing wells in the locality. Surveys conducted in Malawi and Dikwa, Borno State, North East Nigeria [30, 32], are not in agreement with these findings.

In this study, location was associated with urogenital schistosomiasis [C*OR* (95 % *CI*): 1.63 (1.27─2.09)]. Respondents who lived in Dutsin-Ma were twice as likely to be infected [C*OR* (95 % *CI*): 2.31 (1.76─3.03)] compared to those living in Safana. This is consistent with the knowledge that infection becomes prevalent in areas close to dams and the Dutsin-Ma LGA is home to the Zobe Dam, which provides water for irrigation, fishing, recreational activities, and domestic use. Previous studies from Sudan and Nigeria [10, 19] have reported the role location plays in the prevalence of the disease.

Altitude [C*OR* (95 % *CI*): 1.59 (1.14─2.24)] was also found to be associated with infection. Respondents from a higher elevated area had a higher chance of becoming infected with the cercariae of *S. haematobium* probably because such elevations are prone to higher temperatures which favor the hatching of eggs laid by adults into miracidia larvae. Moreover, higher elevated areas easily accumulate water, which in turn harbor the snail intermediate host. Findings in Cross River State, Nigeria, reported a prevalence of 0 % for altitudes above 500 m [33]. In China, people living at the lake-beach level (with hill as a reference point for other elevations) reportedly had the highest odds of getting infected with *S. japonicum*, a closely related species to *S. haematobium* [34].

In the father’s occupation category, “other brown-collar jobs” was associated with urogenital schistosomiasis [C*OR* (95 % *CI*): 1.73(1.19─2.52)], with respondents belonging to this category being about twice [A*OR* (95 % *CI*): 1.97 (1.40─2.77)] as likely to be infected compared to those whose fathers jobs belonged to “the white- collar jobs” category. In the mother’s occupation category, “other brown-collar jobs” was significantly associated with the disease [C*OR* (95 % *CI*): 2.51 (1.29─4.87)], and respondents with mothers in this group are about two times [A*OR* (95 % *CI*):1.89 (1.39─2.56)] as likely to be infected compared to those whose mothers’ jobs belonged to the “white-collar jobs” category. The category of “brown-collar jobs” is closely associated with poverty. Parents of such respondents live below USD 4 per day. In addition to this, the network of potable water sources in the study area is very weak [11]. Consequently, people in this job category cannot exclusively rely on wholesome water sources. Studies conducted in Mali and Yemen has shown that level of income is pertinent to the transmission of urogenital schistosomiasis [35, 36].

## Conclusion

This study showed that Dutsin-Ma and Safana are moderate-risk LGAs for urogenital schistosomiasis. The association of using a combination of borehole, tap, sachet, or well water and acquiring the disease is noteworthy. To this end, further research that seeks to unravel the relationship between the depth/condition (covered or uncovered) of wells and urogenital schistosomiasis is highly recommended. The strong association between the disease and mother’s occupation is of utmost importance and suggests a promising control measure: that is, health education and grassroots mass chemotherapeutic intervention with praziquantel should be directed at mothers. This, like malaria control, could form the basis for the domestic management of schistosomiasis. A Good network of treated pipe-borne water will definitely reduce school children’s contact with infested open water sources.

Non-governmental organizations should distribute molluscicides periodically. A good network including treated pipe-borne water, drainage system, and sewage disposal facilities available should be improved upon.

## Abbreviations

CI, confidence interval; LGA, Local Government Area; OR, odds ratio; SD, standard deviation

## Additional file

Additional file 1: Multilingual abstracts in the six official working languages of the United Nations. (PDF 804 kb)
