# Supplementary material for: Urogenital schistosomiasis and associated determinant factors among senior high school students in the Dutsin-Ma and Safana Local Government Areas of Katsina State, Nigeria
Source: Infect Dis Poverty. 2016 Aug 2;5:69. doi: 10.1186/s40249-016-0158-1 (PMC4969719; doi:10.1186/s40249-016-0158-1)
Supplement: Additional file 1: — Multilingual abstracts in the six official working languages of the United Nations. (PDF 804 kb) [file 40249_2016_158_MOESM1_ESM.pdf]

Translation of the abstract into the six official working languages of the United Nations

داء البلهارسيات البولي التناسلي والعوامل المرتبطة به في صفوف طلاب المدارس الثانوية في المناطق الحكومية المحلية في داستن ما وسافانا في ولاية كاتسينا، نيجيريا

تليلوب إبنازر الثعالبي، عمر الأول، فرنسيس أموونيلا أكنلويي

ملخّص

تقديم عامّ: البلهارسيات البشرية هو مرض طفيلي مزمن ناجم عن الفقر، تتسبّب فيه دودة *cercariae* وهي نوع من المثقوبات *(trematodes)* متناوبة الأجيال من جنس *Schistosoma*. يُعتبر هذا المرض مصدرا رئيسيا للوفاة في 77 بلدا ذوي الدخل المنخفض والمتوسط في المناطق الاستوائية حيث يتعرض 700 مليون شخص للخطر. في محاولة لتوفير معلومات حول انتشار هذا الوباء لتعزيز السيطرة على داء البلهارسيات البولي التناسلي على مستوى نيجيريا، أجرينا هذه الدراسة بهدف التحقيق حول انتشار هذا المرض وحدّته والعوامل المرتبطة بانتشار هذا الوباء.

التقنيات المستخدمة: تم الحصول على البيانات المتعلقة بعوامل الخطر استنادا على 645 طالبا تتراوح أعمارهم بين 12-25 سنة باستخدام مجموعة من الاستبيانات صُممت بأحسن طريقة. تم جمع العينات بين 9:45 و14:00 في زجاجات عالمية. تمّ أخذ 10 ميكرو لتر من كل عينة وتم وضعها بجهاز الطرد المركزي ثمّ فحصها للتأكد من وجود بيض *S. haematobium* باستخدام مجهر (Motic® Light Microscope) نموذج (S-10-P) بعدسة x10. تمّ تسجيل متوسّط حدة الإصابة استنادا على عدد البيض في 10 مل من عينة البول. تم إدخال بيانات الاستبيان في ملفّ Microsoft Excel 2010 وتحليلها باستخدام برنامج Epi Info™ 7. تم تحديد العلاقة بين المتغيرات باستخدام اختبار مربع كاي والانحدار اللوجيستي ثنائي ومتعدد المتغيرات.

النتائج: كان انتشار داء البلهارسيات البولي التناسلي بنسبة 30.54% من بين العدد الإجمالي المدروس، مع متوسط حدة الإصابة 30.27 بيضة/10 مل من البول. كانت نسبة الانتشار ومتوسط حدة الإصابة بالنسبة للذكور أعلى (28.37% و32.21 بيضة/10 مل من البول على التوالي) من تلك المسجلة بالنسبة للإناث (2.17% و5 بيضات/10 مل من البول على التوالي). أنشطة تتطلّب ملامسة الماء ($X^2=29.031, P=0.0000$)، الجنس (ذكر) [$X^2=109.82; P<0.0001$]، الموقع (داستن ما) [$X^2=7.19; P=0.0073$]، عمر المجموعة 18-20 ($X^2=4.819, P=0.0281$)، الآباء الممتهنين لمهنة العامل ($X^2=8.449, P=0.0037$) ومهنة الأمهات ($X^2=9.470, P=0.0021$)، كلّ العوامل ارتبطت بشكل كبير في الإصابة بداء البلهارسيات البولي التناسلي. كان الذكور أكثر عرضة بست مرات للإصابة بدودة *cercariae* من جنس *Schistosoma* مقارنة بالفتيات [A*OR* (95 % *CI*): 6.34 (4.89—8.22)].

الخاتمة: تمّ تصنيف داستن ما وسافانا كمناطق حكومية محلية ذات خطر معتدل للإصابة بداء البلهارسيات البولي التناسلي. إن العلاقة القوية الرابطة بين مهنة الأم والمرض هي أمر بالغ الأهمية وتم اقتراح تدابير رقابة موعودة: توجيه التثقيف الصحي إضافة إلى العلاج الكيميائي باستعمال البرازيكوانتيل بالنسبة للأمهات. ينبغي تحسين الشبكة بما في ذلك، المياه المعالجة التي تنتقل عبر الأنابيب ونظام الصرف الصحي ومرافق الصرف الصحي المتاحة، كما ينبغي استعمال مبيد الرخويات بنسبة معينة للمساعدة على السيطرة على المرض.

Translated from English version into Arabic by Zeineb Trabelsi, through

TRANSLATORS
WITHOUT BORDERS

尼日利亚卡齐纳州 Dutsin-Ma 和 Safana 地方政府区域高中生泌尿生殖道血吸虫病及其相关决定因素

Tolulope Ebenezer Atalabi, Umar Lawal, and Francis Omowonuola Akinluyi

摘要

引言：人体血吸虫病是一种贫困病，也是一种因血吸虫属的复殖吸虫尾蚴所致的慢性寄生虫病，是 77 个热带地区的中、低收入国家发病和死亡的主要原因，威胁着近 7 亿人口。为了给尼日利亚在州水平上提高泌尿生殖道血吸虫病的控制工作提供相关的流行病学信息，我们开展了以调查该病患病率和感染度并鉴定该病流行决定因素为目的的研究。

方法：调查表调查 645 名 12-25 岁的学生相关危险因素，于 09:45 至 14:00 收集尿样。离心 10 µl 尿样，在 Motic® （Binocular）光学显微镜（型号 S-10-P）10 倍物镜下检查埃及血吸虫（*S. haematobium*）虫卵。以每 10 ml 尿样的虫卵数记录平均感染度。调查信息录入 Microsoft Excel 2010，用 Epi Info™ 7 软件分析数据。用卡方检验，双变量和多变量 logistic 回归分析变量间的相互关系。

结果：研究人群的泌尿生殖道血吸虫病患病率是 30.54%，平均感染度为每 10 ml 尿液 30.27 个虫卵。男生的患病率和平均感染度（28.37%和 32.21 虫卵/10 ml 尿液）高于女生（2.17 %和 5 虫卵/10 ml 尿液）。接触水的活动[$X^2=29.031$，$P=0.0000$]、男性[$X^2=109.82$，$P<0.0001$]、地域（Dutsin-Ma）[$X^2=7.19$，$P=0.0073$]、18-20 岁[$X^2=4.819$，$P=0.0281$]、海拔（531─560 m）[$X^2=6.84$，$P=0.0089$]、做棕领工作的父亲[$X^2=8.449$，$P=0.0037$]和母亲[$X^2=9.470$，$P=0.0021$]的职业与泌尿生殖道血吸虫病显著相关。男生感染埃及血吸虫尾蚴的概率是女生的 6 倍[*AOR*（95 % *CI*）：6.34（4.89─8.22）]。
结论： Dutsin-Ma 和 Safana 是泌尿生殖道血吸虫病中度风险地方性政府地区。该病与母亲的职业关系是至关重要的，这意味着针对母亲的健康教育和吡喹酮基层人群化疗是有希望的控制措施。需要改善包括处理的管道水、排水系统和可获得的污水处理设施在内的良好网络。以较高的补贴价率提高灭螺剂有利于该病的控制。

Translated from English version into Chinese by Yin Jian-hai, and edited by Yang Pin

CHINESE CENTER FOR DISEASE CONTROL AND PREVENTION
NATIONAL INSTITUTE OF PARASITIC DISEASES

Schistosomiase urogénitale et facteurs déterminants associés parmi des étudiants du deuxième cycle du secondaire dans les zones de gouvernement local de Dutsin-Ma et de Safana de l'État de Katsina, Nigeria

Tolulope Ebenezer Atalabi, Umar Lawal et Francis Omowonuola Akinluyi

RÉSUMÉ
Contexte : la schistosomiase humaine est une maladie parasitique chronique associée à la pauvreté et causée par les cercaires de trématodes digénétiques du genre *Schistosoma*. Cette maladie constitue une importante source de morbidité et de mortalité dans 77 pays à revenu faible et intermédiaire des tropiques où 700 millions de personnes risquent de la contracter. Afin de fournir des informations épidémiologiques pertinentes dans le but de stimuler la lutte contre la schistosomiase urogénitale au niveau national au Nigeria, nous avons réalisé la présente étude visant à évaluer la prévalence et l'intensité de la maladie et à identifier les facteurs déterminants responsables de son caractère endémique.
Méthodes : des données sur les facteurs de risque ont été obtenues auprès de 645 étudiants âgés de 12 à 25 ans à l'aide de questionnaires élaborés de manière appropriée. Des échantillons ont été collectés entre 09h45 et 14h00 dans des récipients universels. Chaque échantillon de 10 µl centrifugé a été examiné afin d'y identifier la présence d'œufs de *S. haematobium* à l'aide d'un microscope optique (binoculaire) Motic® (modèle S-10-P) avec un objectif x10. L'intensité moyenne de l'infection était exprimée en nombre d'œufs par 10 ml d'urine prélevée. Les données d'étude ont été saisies dans Microsoft Excel 2010 et analysées à l'aide du programme Epi Info™ 7. Des associations parmi les variables ont été déterminées à l'aide du test de chi carré et d'une analyse de régression logistique bivariée et multivariée.
Résultats : la prévalence de la schistosomiase urogénitale atteignait 30,54 % parmi la population à l'étude et l'intensité moyenne de l'infection s'élevait à 30,27 œufs/10 ml d'urine. La prévalence et l'intensité moyenne de l'infection étaient plus élevées chez les hommes (28,37 % et 32,21 œufs/10 ml d'urine) que chez les femmes (2,17 % et 5 œufs/10 ml d'urine). Nous avons déterminé que les activités en contact avec l'eau ($X^2=29{,}031$, $P=0{,}0000$), le sexe (masculin) [$X^2=109{,}82$; $P<0{,}0001$], la localisation (Dutsin─Ma) [$X^2=7{,}19$; $P=0{,}0073$], le fait d'appartenir au groupe d'âge des 18-20 ans ($X^2=4{,}819$, $P=0{,}0281$), l'altitude (531─560 m) [$X^2=6{,}84$, $p=0{,}0089$], l'exercice d'autres professions à faible revenu par le père ($X^2=8{,}449$, $P=0{,}0037$) et la profession de la mère ($X^2=9{,}470$, $P=0{,}0021$) présentaient une forte association avec la schistosomiase urogénitale. Les jeunes hommes avaient six fois plus de chance de contracter une infection due à des cercaires de type *S. haematobium* que les jeunes femmes [*AOR* (*IC* à 95 %) : 6,34 (4,89─8,22)].
Conclusions : les zones de gouvernement local de Dutsin-Ma et de Safana ont été classées comme présentant un risque modéré d'épidémie de schistosomiase urogénitale. La forte association de la maladie et de l'activité de la mère est d'une importance primordiale et suggère une mesure de lutte prometteuse qui consisterait à faire profiter les mères des campagnes de sensibilisation à la

santé ainsi que des interventions chimiothérapeutiques de masse communautaires reposant sur l'administration de praziquantel. Il convient d'améliorer la qualité du réseau couvrant le traitement de l'eau courante, le système de drainage et les installations d'évacuation des eaux usées. Il serait nécessaire de mettre des molluscicides à disposition à des tarifs fortement subventionnés afin de contribuer à la lutte contre la maladie.

Translated from English version into French by Eric Ragu, through

TRANSLATORS WITHOUT BORDERS

Мочеполовой шистосомоз и связанные с определяющими факторами среди старшеклассников в территории местного управления Штата Катсина, Нигерия

Толулопе Эбенезер Аталаби, Умар Лаваль, и Фрэнсис Омоуонуола Акинлуий

АННОТАЦИЯ

Цель: Человек шистосомоз является хроническим паразитарным (инвазионным) заболеванием бедности, вызванное церкарием (личиночная стадия дигенетических сосальщиков) дигенетических трематодами рода шистосомы.
Заболевание является основным источником заболеваемости и смертности в 77 странах с низким и средним уровнем дохода в тропиках, где 700 миллионов человек подвергаются риску. В стремлении обеспечить соответствующую эпидемиологическую информацию для повышения контроля над урогенитального шистосомоза на государственном уровне в Нигерии, мы провели это исследование с целью изучения распространенности и интенсивности этого заболевания, а также определяющих факторов, ответственных за его распространенностью.

Методы: Данные о факторах риска были получены всего от 645 студентов в возрасте 12─25 лет с использованием хорошо разработанных вопросников. Образцы были собраны между 09:45 и 14:00 в универсальных бутылках. Каждый 10µl центрифугированный образец исследованы на яйца S. haematobium с использованием Motic® (бинокулярный) световой микроскоп (модель S-10-P) с объективом x10. Среднюю интенсивность инфекции регистрировали как количество яиц на 10 мл пробы мочи. Данные обследования были введены в Microsoft Excel 2010 и анализированы с использованием Epi Info ™ 7. Логическая взаимосвязь между переменными определяли с помощью теста хи-квадрат и двумерных и многомерных логистических регрессий.

Результаты: Распространенность мочеполового шистосомоза была 30,54% среди исследуемой популяции со средней интенсивностью зараженности яиц 30.27 / 10 мл мочи. Распространенность и средняя интенсивность были выше у мужчин (28,37% и 32,21 яиц / 10 мл мочи соответственно), чем у женщин (2,17% и 5 яиц / 10 мл мочи, соответственно). Активность контакта с источником заражения-водой (X^2 = 29,031, *P* = 0,0000), пол (мужской) [X^2 = 109,82; *P*<0.0001], место нахождения (Дутсин─Ма) [X^2 = 7,19; *P* = 0,0073], возрастная группа 18-20 (X^2 = 4,819, *P* = 0,0281), высота над уровнем моря (531─560 м) [X^2 = 6,84, *P* = 0,0089], отцы делают другие черные работы (X^2 = 8,449, *P* = 0,0037) и род занятий матерей (X^2 = 9,470, *P* = 0,0021) были обнаружены в значительной степени связаны с мочеполовой шистосомоз. У мальчиков были в шесть раз больше вероятностей заражения с овцеркарием S. haematobium по сравнению с девочками [A*OR* (95% ДИ): 6,34 (4.89─8.22)].

Выводы: Дутсин-Ма и Сафана были классифицированы как территория местного управления с умеренном риском мочеполового шистосомоза. Очень близкая связь между болезнью и занятией матери имеет огромное значение, и предлагает перспективные контрольные меры: то есть, направление медико-санитарного просвещения, а также низового массы химиотерапевтической вмешательство с празиквантела на матерей. Доступная хорошая сеть в том числе обработанной воды из трубы, дренажной системы и очистных сооружений должны быть улучшены. Моллюскициды должна предоставляться на

весьма заниженным тарифам, чтобы помочь борьбе с этой болезнью.

Translated from English version into Russian by Turdimurot Rakhmonov, through

Esquistosomiasis urogenital y factores determinantes relacionados en estudiantes de secundaria de las áreas de gobierno local Dustin-Ma y Safana, en el estado de Katsina, Nigeria.

Tolulope Ebenezer Atalabi, Umar Lawal, and Francis Omowonuola Akinluyi

Resumen

Introducción: La esquistosomiasis humana es una enfermedad parasitaria crónica relacionada con la pobreza provocada por cercadias de trematodos digeneos del género *Schistosoma.* Esta enfermedad es una importante causa de morbilidad y mortalidad en 77 países de ingresos bajos y medios en los trópicos, donde 700 millones de personas están en riesgo. En un intento por proporcionar información epidemiológica relevante para mejorar el control de la esquistosomiasis urogenital a nivel estatal en Nigeria, llevamos a cabo este estudio con el objetivo de investigar la prevalencia e intensidad de la enfermedad y los factores determinantes detrás de su carácter endémico.

Métodos: Por medio de cuestionarios bien diseñados, se obtuvieron datos sobre los factores de riesgo de un total de 645 estudiantes de entre 12 y 25 años. Ente las 09:45 y las 14:00, se recolectaron muestras en frascos universales. Se examinó cada una de las muestras centrifugadas de 10µl para detectar huevos de *S. haematobium* con un microscopio binocular de luz Motic® (modelo S-10-P) con objetivo x10. Se registro la intensidad media de infección de acuerdo a la cantidad de huevos cada 10ml de muestra de orina. Los datos obtenidos fueron ingresados en Microsoft Excel 2010 y analizados con Epi Info™ 7. Se determinaron las asociaciones entre variables por medio del la prueba de chi-cuadrado y de regresiones logísticas bi-variables y multi-variables.

Resultados: La prevalencia de esquistosomiasis urogenital entre la población estudiada fue de un 30,54%, con una intensidad media de infección de 30,27 huevos/10 ml de orina. La prevalencia y la intensidad media fueron mayores en hombres (28,37% y 32,21 huevos/10 ml de orina respectivamente) que en mujeres (2,17% y 5 huevos/10 ml of orina respectivamente). Se descubrió que las actividades de contacto con el agua ($X^2=29.031, P=0.0000$), el sexo (masculino) [$X^2=109.82; P<0.0001$], la ubicación (Dutsin─Ma) [$X^2=7.19; P=0.0073$], el grupo etario de entre 18 y 20 ($X^2=4.819, P=0.0281$), la altitud (531─560 m) [$X^2=6.84, p=0.0089$], el hecho de que los padres tuvieran diversos trabajos manuales poco remunerados ($X^2=8.449, P=0.0037$) y la ocupación de las madres ($X^2=9.470, P=0.0021$) están asociados de manera significativa con la esquistosomiasis urogenital. Era seis veces más probable que estuvieran infectados con la cercadia del *S. haematobium* los varones que las chicas [A*OR* (95 % *CI*): 6.34 (4.89─8.22)].

Conclusiones: Se clasificó a Dutsin-Ma y Safana como áreas de gobierno local con riesgo moderado de esquistosomiasis urogenital. La fuerte relación entre la enfermedad y la ocupación de la madre es de suma importancia y representa una medida de control prometedora: enfocar la educación sanitaria y las intervenciones quimioterapéuticas de base con praziquantel en las madres. También se debería desarrollar una buena red de suministro de agua tratada, un sistema de drenaje e instalaciones de eliminación de aguas residuales. Por último, se deberían proveer y subsidiar molusquicidas para ayudar a controlar la enfermedad.

Translated from English version into Spanish by Mpgorgone, through
